# Technetium-99m Radiopharmaceuticals for Ideal Myocardial Perfusion Imaging: Lost and Found Opportunities

**DOI:** 10.3390/molecules27041188

**Published:** 2022-02-10

**Authors:** Alessandra Boschi, Licia Uccelli, Lorenza Marvelli, Corrado Cittanti, Melchiore Giganti, Petra Martini

**Affiliations:** 1 Department of Chemical, Pharmaceutical and Agricultural Sciences, University of Ferrara, Via L. Borsari, 46-44121 Ferrara, Italy; lorenza.marvelli@unife.it; 2 Department of Translational Medicine, University of Ferrara, Via Fossato di Mortara, 70 c/o Viale Eliporto, 46-44121 Ferrara, Italy; licia.uccelli@unife.it (L.U.); corrado.cittanti@unife.it (C.C.); melchiore.giganti@unife.it (M.G.); 3 Department of Environmental and Prevention Sciences, University of Ferrara, Via L. Borsari, 46-44121 Ferrara, Italy; petra.martini@unife.it

**Keywords:** technetium-99m, myocardial perfusion agents, ideal tracers

## Abstract

The favorable nuclear properties in combination with the rich coordination chemistry make technetium-99m the radioisotope of choice for the development of myocardial perfusion tracers. In the early 1980s, [^99m^Tc]Tc-Sestamibi, [^99m^Tc]Tc-Tetrofosmin, and [^99m^Tc]Tc-Teboroxime were approved as commercial radiopharmaceuticals for myocardial perfusion imaging in nuclear cardiology. Despite its peculiar properties, the clinical use of [^99m^Tc]Tc-Teboroxime was quickly abandoned due to its rapid myocardial washout. Despite their widespread clinical applications, both [^99m^Tc]Tc-Sestamibi and [^99m^Tc]Tc-Tetrofosmin do not meet the requirements of an ideal perfusion imaging agent due to their relatively low first-pass extraction fraction and high liver absorption. An ideal radiotracer for myocardial perfusion imaging should have a high myocardial uptake; a high and stable target-to-background ratio with low uptake in the lungs, liver, stomach during the image acquisition period; a high first-pass myocardial extraction fraction and very rapid blood clearance; and a linear relationship between radiotracer myocardial uptake and coronary blood flow. Although it is difficult to reconcile all these properties in a single tracer, scientific research in the field has always channeled its efforts in the development of molecules that are able to meet the characteristics of ideality as much as possible. This short review summarizes the developments in ^99m^Tc myocardial perfusion tracers, which are able to fulfill hitherto unmet medical needs and serve a large population of patients with heart disease, and underlines their strengths and weaknesses, the lost and found opportunities thanks to the developments of the new ultrafast SPECT technologies.

## 1. Introduction

Heart disease is the leading cause of death worldwide. Among heart diseases, the progressive narrowing of the coronary artery due to atherosclerotic deposits, known as coronary artery disease (CAD), is a major cause of early and permanent disability. In order to be able to administer appropriate therapeutic regimes before irreversible myocardial damage occurs, early detection is essential. In particular, the precise measurement of regional blood flow has significant clinical importance in the identification of ischemia, in the definition of the extent and severity of the disease, in the assessment of the viability of the myocardium, in monitoring the effects of treatment, and in determining the need for medical and surgical interventions.

Nuclear cardiology plays a key role in the management of CAD patients [1,2,3,4,5,6,7,8,9,10]. It employs small molecules containing a gamma emitter radionuclide for single-photon emission computed tomography (SPECT) or a positron emitter radionuclide for positron emission tomography (PET). For myocardial perfusion imaging, a quantity of radiotracer proportional to the regional blood flow should be located into the cardiac muscle in order to evaluate areas with reduced blood flow due to ischemia. Therefore, in the patient with CAD, the area of reduced blood flow is observed based on a reduced fixation of the radio-indicator. In the case of ischemia, the area of reduced absorption is worse under stress than under rest conditions. Therefore, information obtained during image perfusion studies can be used not only to identify CAD, but also to present prognostic information about the probability of a severe adverse cardiac event, such as myocardial infarction or related cardiac death that may occur.

Currently, three SPECT blood-flow markers are clinically available and widely used: [^201^Tl]TlCl, [^99m^Tc]Tc-Sestamibi, and [^99m^Tc]Tc-Tetrofosmin. [^201^Tl]TlCl was introduced in the mid-1970s and involved a breakthrough in the widespread clinical use of myocardial perfusion with a profound impact on therapeutic decision-making in CAD patients over the past four decades [11]. Thallium-201 is a potassium analog and is extracted actively by cell membrane Na/K pumps on the first pass through the coronary vasculature. It has a high peak myocardial concentration at 10 min after injection and the advantage of infarction detection within 6 h after the onset of symptoms [11]. However, this radionuclide has several limitations that result in poor image captures, such as a long half-life (t_1/2_ = 73 h), attenuation artifacts caused by the limited abundance of gamma photons, and lower counting rates caused by dose constraints, so much so that the search for better imaging myocardial perfusion agents began immediately after its clinical introduction [12,13]. Another limitation of [^201^Tl]TlCl is the dynamic nature of its kinetics, namely its redistribution, which implies that imaging should begin immediately after injection, which is unsuitable condition for patients with acute myocardial infarction or in the regulation of chest pain centers, where immediate imaging cannot be performed.

Compared to thallium-201, technetium-99m produces relatively high-energy photons (~140 KeV) and can be used at high doses due to its short half-life (t_1/2_ = 6.01 h). The favorable nuclear properties, in combination with the rich coordination chemistry [14], make technetium-99m the isotope of choice for the development of radiotracers for myocardial perfusion studies [14]. In the early 1980s, intensive efforts were made to develop [^99m^Tc]-radiopharmaceuticals [15]. Consequently, [^99m^Tc]Tc-Sestamibi, [^99m^Tc]Tc-Tetrofosmin, and [^99m^Tc]Tc-Teboroxime were approved as commercial radiopharmaceuticals for myocardial perfusion imaging in nuclear cardiology. These [^99m^Tc]-tracers are highly lipophilic with a cationic or neutral charge and are excreted through the hepatobiliary system due to their elevated lipophilia. Among these tracers, [^99m^Tc]Tc-Sestamibi and [^99m^Tc]Tc-Tetrofosmin have experienced widespread prolonged clinical use while [^99m^Tc]Tc-Teboroxime, despite its initial approval by the FDA, is not in widespread clinical use. Due to the high initial absorption and rapid washout, the image acquisition with [^99m^Tc]Tc-Teboroxime must take place within 2 min of injection, which, before the introduction of the cadmium-zinc-telluride (CZT) semiconductor detectors for SPECT-CT instrumentation, was technically demanding.

Despite their widespread clinical applications, both [^99m^Tc]Tc-Sestamibi and [^99m^Tc]Tc-Tetrofosmin do not meet the requirements of an ideal perfusion imaging agent due to their relatively low first-pass extraction fraction and high liver absorption [16].

An ideal radiotracer for myocardial perfusion imaging (MPI) should have:

(a) High myocardial uptake with minimal or absent myocardial redistribution;
(b) High and stable target-to-background ratio with low uptake in the lungs, liver, stomach, and gut during the image acquisition period. In particular, absorption into the liver and lungs should be minimal, so that diagnostic useful images can be obtained within 30 min after injection. The intense absorption of the liver and the subsequent visualization, through radioactive bile, of the gut and stomach makes it difficult to interpret cardiac activity in the lower and left ventricular walls;
(c) High first-pass myocardial extraction fraction and very rapid blood clearance. These are both fundamental characteristics for evaluating the kinetics of entry of the radiopharmaceutical into the myocyte and the relative removal from the bloodstream. A low extraction fraction of the first passage often leads to an inaccurate determination of blood flow characteristics;
(d) A linear relationship between radiotracer myocardial uptake and coronary blood flow.

The medical need to have new radiotracers with a faster liver clearance and a better extraction fraction of the first step, compared to [^99m^Tc]Tc-Sestamibi and [^99m^Tc]Tc-Tetrofosmin, has given a big boost to research in the last 15–20 years. In this particular field, coordination chemistry plays a fundamental role in the development of new [^99m^Tc]-radiotracers, where the chemical structure of developed molecules determines particular biodistribution properties and, hence, their diagnostic potential. We should not forget that [^99m^Tc]-radiopharmaceuticals developed for myocardial perfusion have all the characteristics of coordination complexes and their localization is closely linked to their chemical structure, size, charge, and lipophilia, resulting from the coordination of a special ligand with the metal core.

This short review summarizes the developments in technetium-99m myocardial perfusion tracers able to fulfill hitherto unmet medical needs and serve a large population of patients with heart disease. Their chemical characteristics, preparation methods, strengths and weaknesses, and the opportunities lost and found thanks to the developments of the new ultrafast SPECT technologies will be described.

## 2. Technetium-99m Radiopharmaceuticals for MPI in Clinical Use

**Figure 1:**
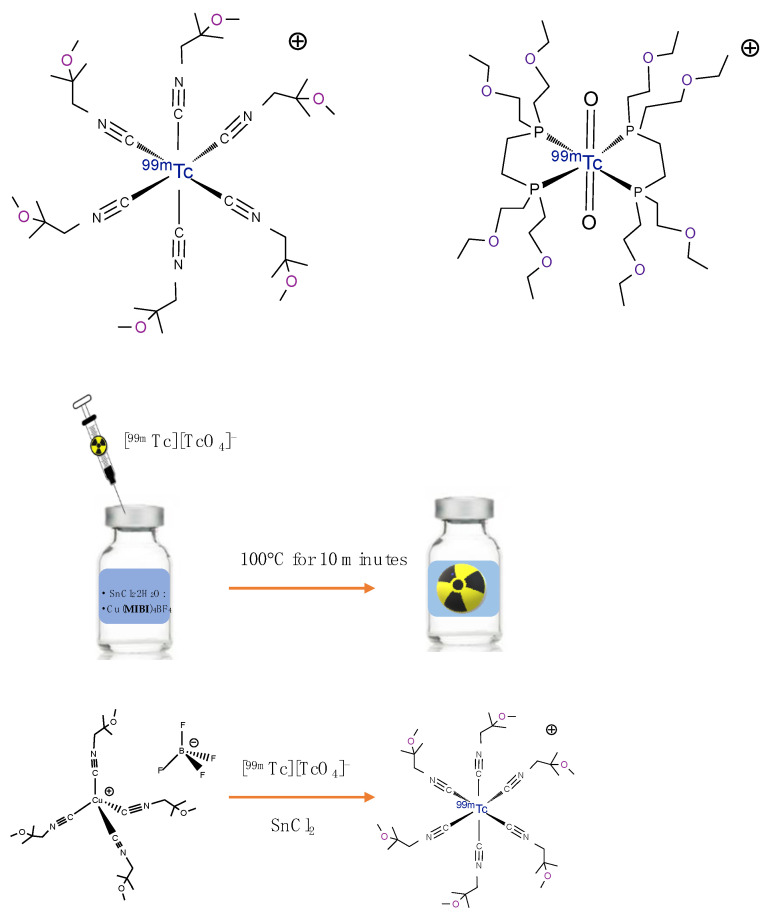
Chemical structure of [^99m^Tc]Tc-Sestamibi (**left**) and [^99m^Tc]Tc-Tetrofosmin (**right**), and a schematic representation of the preparation of [^99m^Tc]Tc-Sestamibi.

To date, [^99m^Tc]Tc-Sestamibi and [^99m^Tc]Tc-Tetrofosmin (Figure 1) are the most used ^99m^Tc-radiopharmaceuticals for the study of ischemic heart disease. The development of these radiopharmaceuticals is due to their peculiar chemical–physical properties as well as the ease of preparation (Figure 1). [^99m^Tc]Tc-Sestamibi is a mono-cationic lipophilic complex formed by the coordination of six identical 2-methoxyisobutylsonitrile (MIBI) ligands to the central technetium atom in the oxidation state +1. It is easily prepared through the addition of generator eluted [^99m^Tc]NaTcO_4_ to a commercial kit containing all the reagents in freeze-dried form. Due to the high volatility, the MIBI ligand is present in the lyophilized formulation as a copper salt, Cu(MIBI)_4_BF_4_, in order to avoid its loss during the process of lyophilization for the preparation of the “cold” kit. To facilitate the exchange reaction of isonitrile ligands, which need to remove the copper complex to coordinate the technetium, after the addition of [^99m^Tc]NaTcO_4_, the kit is heated to 100 °C for 10 min.

The accumulation of cationic radiopharmaceuticals, such as [^99m^Tc]Tc-Sestamibi and [^99m^Tc]Tc-Tetrofosmin in the myocytes, is related to their ability to follow progressive electronegative transmembrane potential from the blood to the mitochondrial matrix, in which the negative attractive charges are relatively more present. Its intracellular passage is permanent and redistribution is negligible [17]. As a result, separate rest and stress images are required for the detection of reversible stress-related perfusion defects.

[^99m^Tc]Tc-Sestamibi myocardial uptake is at maximum 1 min after injection, and <5% activity is in the blood 5 min post-injection. Its extraction coefficient (65%) is considerably lower than that of thallium-201, which has the highest myocardial coefficient (85%) of both the [^99m^Tc]-labelled blood flow markers. The heart uptake is 1% of the injected dose after rest injection, and 1.4% after exercise injection at 1 h post intravenous injection, respectively. This radiopharmaceutical rapidly clears from the blood pool [18].

[^99m^Tc]Tc-Sestamibi is mainly excreted by the hepatobiliary system, with a small renal excretion. The highest radiation doses are in the upper large intestine, small intestine, and gall bladder. With a 1.11 GBq injection, the upper large intestines receive 46 and 47 mGy, respectively, with the exercise and rest injections, whereas the whole-body radiation dose is 4.9 mGy in the exercise condition and 4.6 mGy for the rest studies [18].

[^99m^Tc]Tc-Tetrofosmin consists of two identical di-phosphine ligands coordinated to the trans-technetium di-oxo core [O=Tc=O]^+^, as shown in Figure 1, to give the lipophilic di-oxo-monocation [^99m^Tc][Tc(tetrofosmin)_2_O_2_]^+^ complex in which technetium is in the V oxidation state. Unlike [^99m^Tc]Tc-Sestamibi, this radiopharmaceutical is prepared at room temperature in 15 min after the addition of the generator eluted [^99m^Tc]NaTcO_4_ to the kit, containing SnCl_2_x 2H_2_O, the ligand Tetrofosmin, and some excipients in freeze-dried form. The presence of tin, also common to the formulation of [^99m^Tc]Tc-Sestamibi, is required to reduce the technetium (VII) oxidation state in pertechnetate. 

[^99m^Tc]Tc-Tetrofosmin has a lower myocardial extraction coefficient (54%) than [^99m^Tc]Tc-Sestamibi. It allows for the visualization of the myocardium already in the images acquired 5 min after injection, as almost 1.2% of the administered activity is absorbed by the myocardium both at rest and in exercise protocols, with good retention and rapid blood clearance, with less than 5% blood pool activity at 10 min after injection. The liver uptake decreases rapidly from 7.5% ± 1.7% at 5 min, to 2.1% ± 1.0% at 1 h after the rest injection, exhibiting the fastest liver washout among the available commercial agents. [^99m^Tc]Tc-Tetrofosmin has a relatively low lung, hepatic, gastrointestinal, and splenic uptake after stress and rest injections, and rapid clearance from these organs [19]. For this reason, rest images can be obtained 30 min after the injection, and the stress images can be acquired from 5 to 10 min after the radiopharmaceutical injection, with convenient 1 day or even shorter stress-rest imaging protocol application. The clearance by renal and fecal routes is similar [20], where about 72% and 67% of the administered activity is excreted from the body by 48 h after the rest and exercise injections, respectively. The excretory organs received the highest radiation dose. The estimated whole-body dose after 1.11 GBq [^99m^Tc]Tc-Tetrofosmin administration was 10 mGy in the rest studies and 8 mGy after exercise. The gall bladder received the highest individual organ dose (from 37 to 54 mGy). 

Despite the commercial agents, [^99m^Tc]Tc-Sestamibi and [^99m^Tc]Tc-Tetrofosmin have good image properties; they do not have all the characteristics commonly considered typical of an ideal tracer, which are the high first-pass extraction, fast liver washout, and linear relationship between blood flow and tracer uptake in the myocardium. Even after the spread of these tracers, nuclear cardiology never stopped searching for an ideal imaging agent for cardiac perfusion, although it is commonly acknowledged that it is not always possible to accommodate all these features in the same ^99m^Tc-complex. For example, it seems quite difficult to combine in the same ^99m^Tc-compound a high first-pass extraction and fast liver washout.

The following paragraphs describe the different classes of experimental myocardial perfusion agents studied to develop new radiotracers that satisfy the unmet medical need.

## 3. Technetium-99m Teboroxime

**Figure 2:**
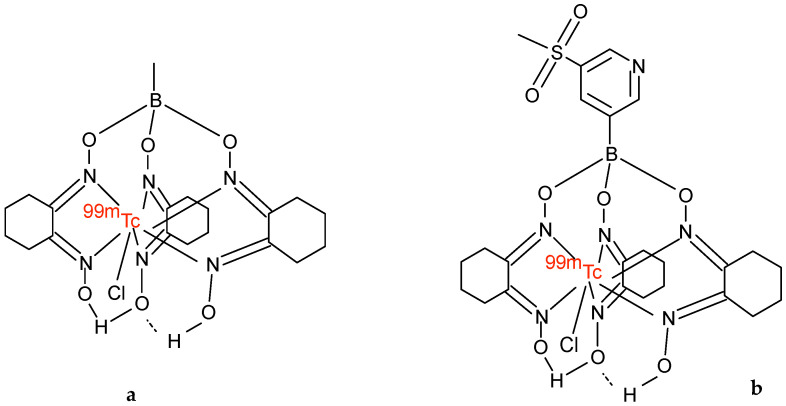
Chemical structure of (**a**) [^99m^Tc]Tc-Teboroxime, (**b**) [^99m^Tc]Tc-3SPboroxime.

[^99m^Tc]Tc-Teboroxime was approved in the early 1990s by the Food and Drug Administration (FDA) for clinical use and, similar to [^99m^Tc]Tc-Sestamibi, became commercially available at the end of 1990 [21]. [^99m^Tc]Tc-teboroxime (bis(1,2-cyclohexanedione dioxi- mato(1-)-0)-(1,2-cyclohexane-dione-ioximato(2-)-0) methyl borato(2-)-N,N′,N′′,N′′′,N′′′′,N′′′′′)-chloro-technetium (Figure 2) is a neutral lipophilic agent that is chemically very different from [^99m^Tc]Tc-Sestamibi or [^99m^Tc]Tc-Tetrofosmin, with a molecular size larger than [^201^Tl]Tl and smaller than [^99m^Tc]Tc-Sestamibi. It is a member of the boronic acid adducts of technetium dioxime complexes (BATOs). These are seven coordinate technetium dioxime complexes having a boronic adduct capped at the end of the molecule.

After intravenous administration, the myocardial absorption of [^99m^Tc]Tc-Teboroxime is very fast, with excellent myocardial visualization at 1–2 min after injection [22,23,24,25]. Myocardial clearance, however, is very fast; with two-thirds of myocardial activity washout within 4 min, [^99m^Tc]Tc-Teboroxime spreads rapidly through the cell membrane due to its neutral and highly lipophilic characteristics and also binds to red blood cells, which in part explains the rapid washout from the heart [26]. Blood clearance is rapid, with only 9.5% of the remaining dose circulating at 15 min after injection. Elimination is predominantly hepatic. Liver absorption, initially low, increases over time with maximum activity at about 5 min after injection. The liver half-life of about 1 to 1.5 h differs from that of [^99m^Tc]Tc-Sestamibi, to indicate that absorption and excretion mechanisms are different. This tracer has the highest first-pass myocardial extraction of all myocardial perfusion radiopharmaceuticals and shows better linearity (0–4.5 mL/min/g) between myocardial absorption and myocardial blood flow than that of [^99m^Tc]Tc-Sestamibi and [^99m^Tc]Tc-Tetrofosmin [27]. Due to its rapid cardiac washout, imaging should be finished within 5 to 6 min of its injection. This is difficult to achieve in most clinical studies and its clinical use has been rapidly abandoned.

A new [^99m^Tc]Tc-Teboroxime derivative was recently studied to increase retention in the myocardium. Xi et al. [28] reported studies on Sprague Dawley rats of [^99m^Tc]Tc-3SPboroxime, [^99m^Tc]TcCl(CDO)(CDOH)_2_B-3SP)3SP-B(OH)_2_=3-(methylsulfonyl)pyridineboronic acid, another member of the adduct boronic acid complex [^99m^Tc]Tc (III). This compound has the same structural characteristics as the original [^99m^Tc]Tc-Teboroxime complex, except for the replacement of the methyl group added to the boron cap by a sulfonyl group [29] (Figure 2). [^99m^Tc]Tc-3SPboroxime was obtained by the reaction between the metal with cyclohexanedione dioxime assisted by 3-(methylsulfonyl)phenylboronic acid, in the presence of SnCl_2_ and [^99m^Tc][TcO_4_]^−^. The authors compared this tracer with [^99m^Tc]Tc-Teboroxime and [^99m^Tc]Tc-Sestamibi, in order to study the myocardial absorption, heart–liver ratio, and K1 and K2 values for the compartment model in the oscillations of normal and acute myocardial infarction. Their results showed that initial uptake was comparable to that of [^99m^Tc]Tc-Teboroxime, but significantly higher than that of [^99m^Tc]Tc-Sestamibi. Moreover, the heart/liver ratio was higher than that of [^99m^Tc]Tc-Teboroxime 15 min after injection. Its first-pass myocardial extraction fraction was higher than that of [^99m^Tc]Tc-Sestamibi, the K1 [^99m^Tc]Tc-3SPboroxime value was significantly higher, and the K2 values were significantly lower than that of [^99m^Tc]Tc-Teboroxime, both at rest and after the administration of dipyridamole, indicating a longer myocardial retention time for [^99m^Tc]Tc-3SPboroxime. The authors obtained high-quality SPECT images in the first 15 min after injection, using a cadmium zinc telluride CZT-SPECT camera.

Pig model studies confirm that [^99m^Tc]Tc-3SPboroxime shows high initial heart uptake, comparable to that of [^99m^Tc]Tc-Teboroxime, but myocardial clearance is significantly delayed [28,30]. The high initial heart uptake, combined with longer myocardial retention and high heart/background ratios, suggests that this compound may become an attractive candidate for future clinical application by means of the new ultrafast SPECT detectors. 

Obviously, although these are very interesting results, further studies will be needed to validate the potential use of [^99m^Tc]Tc-3SPboroxime in myocardial blood flow quantification and biodistribution data obtained in humans. 

The properties of selected ^99m^Tc-labeled MPI agents are summarized in Table 1.

## 4. Technetium-99m Radiopharmaceuticals for MPI Based on the Nitride Core

### 4.1. [^99m^Tc][TcN(NOEt)_2_]

The bis(N-ethoxy, N-ethyl dithiocarbamate) nitrido [^99m^Tc]Tc(V), known as [^99m^Tc][TcN(NOEt)_2_], is a lipophilic neutral myocardial perfusion imaging agent belonging to the ^99m^Tc nitrido dithiocarbamates class, symmetrical neutral disubstituted compounds. These complexes are characterized by the coordination of ligands exhibiting (S,S^-^) a donor array to the [^99m^Tc]TcN core [31]. This radiopharmaceutical is prepared through a two-vial lyophilized kit formulation; in the first step ^99m^Tc-pertechnetate is added to a vial containing succinic dihydrazide (SDH), SnCl_2_ × 2H_2_O, and 1,2-propane-N,N,N′,N′-tetraacetic acid (DTPA). The resulting mixture is kept at room temperature for 15 min. The intermediate compound bearing the [Tc ≡N]^2+^ core is then mixed with the sodium salt of N-ethoxy, N-ethyl dithiocarbamate, and β-methyl-cyclodextrin, which serves as a solubilizing agent, to obtain a neutral [^99m^Tc][TcN(NOEt)_2_] compound. Radiochemical purity is checked by thin-layer chromatography [32]. Its unique imaging properties are remarkable and, for this reason, it underwent extensive clinical evaluation in patients. The trials were conducted on healthy volunteers showed imaging properties and kinetic behavior much more similar to those of thallium-201 than other ^99m^Tc-monocationic complexes: the cardiac absorption of [^99m^Tc][TcN(NOEt)_2_] is about 4% of the injected activity at 5 min after injection, and the leaching of activity from myocardial tissue has a half-life of about 3 h [33]. It also shows a high first-pass extraction ranging from 80% to 90% and a good correlation with coronary blood flow. It has been established that myocardial absorption is proportional to the blood flow of the heart at low and medium flow rates, becoming about 80% at higher flows [34,35]. The redistribution is favored by the absence of a specific intracellular link [36] and by the relatively high level of a radiotracer in circulating blood. These properties allow the tracer to move freely through the vital areas of the heart and to spread slowly from high activity areas to regions of low activity, such as the myocardial tissue normally perfused against ischemic areas. For all these reasons, [^99m^Tc][TcN(NOEt)_2_] allows the same approach as the ^201^Tl stress–rest studies to be conducted, using, therefore, a single injection exploiting the redistribution mechanism. Furthermore, this modality, which represents a considerable advantage in terms of dosimetry with respect to ^201^Tl, appears to be more favorable also with respect to cationic radiotracers, which require two separate administrations [37]. Despite these unique properties that make the lipophilic and neutral [^99m^Tc][TcN(NOEt)_2_] complex a pure myocardial blood flow tracer, the transient lung absorption of the tracer during the first minutes post-injection and its persistent liver uptake are important drawbacks. For these reasons, this technetium-99m radiopharmaceutical has not been approved for clinical use.

### 4.2. [^99m^Tc]TcN-PNP Monocationic Compounds

**Figure 3:**
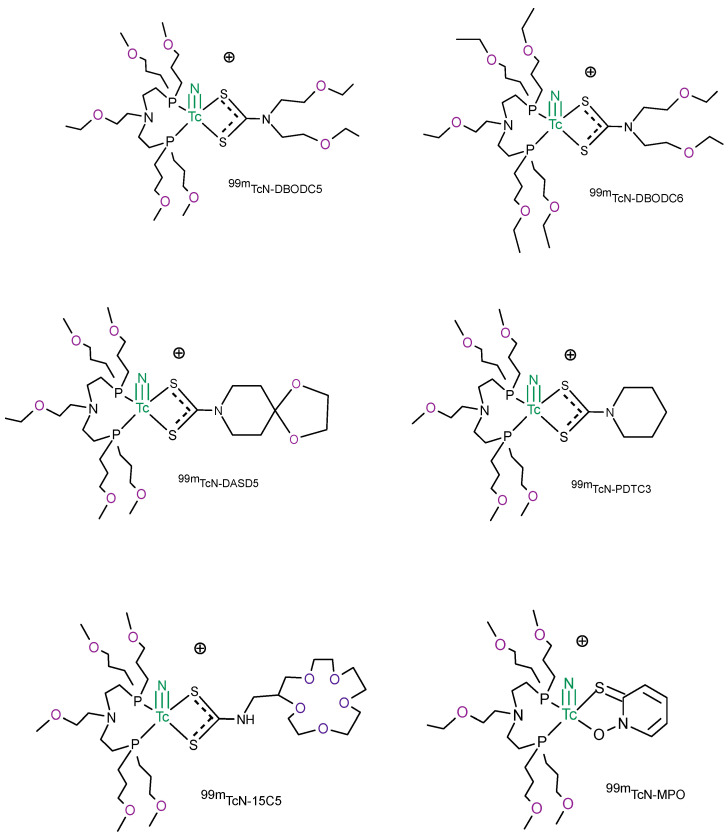
Chemical structures of the most representative [^99m^Tc][TcN-PNP] monocationic myocardial perfusion imaging compounds.

After the development of the [^99m^Tc][TcN(NOEt)_2_], some asymmetrical monocationic compounds containing the [^99m^Tc]TcN multiple bond group [Tc≡N]^2+^, a bidentate dithiocarbamate (DTC) and a PNP type bisphosphine, were investigated as potential ^99m^Tc myocardial perfusion tracers. This class of complexes has the general formula [^99m^Tc][TcN(DTC)(PNP)]^+^, where the nitride core is coordinated to a bis-phosphinoamine ligand, and to one monoanionic dithiocarbamate chelate bearing linear, alicyclic, or crown etheric arms (Figure 3). The nitrogen atom in the bis-phosphinoamine ligand is reported to provide stabilization for the technetium nitride core due to the weak interaction with the metal [38,39]. The preparation of these compounds is accomplished in a physiological solution through a two-vial lyophilized kit formulation; in the first step, ^99m^Tc-pertechnetate is added to a vial containing succinic dihydrazide (SDH), SnCl_2_ × 2H_2_O, and 2,2′,2′′,2′′′-(ethane-1,2-diyldinitrilo)tetra acetic acid (EDTA) to form the technetium nitride core. The intermediate compound bearing the [Tc≡N]^2+^ core is then simultaneously mixed with PNP and DTC ligands. The combination of PNP bisphosphine and DTC results in the high radiochemical purity of cationic 99mTc-nitrido complexes [38,39].

This class of agents showed improved pharmacokinetic profiles in rats compared to those commercial agents, [^99m^Tc]Tc-Sestamibi and [^99m^Tc]Tc-Tetrofosmin, with high initial and persistent heart uptake, rapid blood clearance, and elimination from non-target tissues.

The results from animal studies evaluated in Sprague Dawley rats show that both dithiocarbamate and bisphosphines ligands have a notable impact on the biodistribution properties and excretion kinetics of cationic [^99m^Tc][TcN(DTC)(PNP)]^+^complexes [40,41]. [^99m^Tc][TcN-DBODC5] shows a high heart uptake with more than 2 h of retention in the rat myocardium. The liver uptake is almost completely eliminated after 2 h from the injection, and the heart to liver ratio is ten times better than that of [99mTc]Tc-Sestamibi [41]. Its first-pass extraction is between that of [^99m^Tc]Tc-Sestamibi and [^99m^Tc]Tc-Tetrofosmin [42]. These features allow clear images of the heart as early as 15 min post-injection and, of this category of complexes, only [99mTc][TcN-DBODC5] have undergone clinical evaluation.

On the contrary, the compound [^99m^Tc][TcN-DBODC6] shows a lower uptake in the heart and prolonged retention in lungs than [99mTc][TcN-DBODC5], due to the higher lipophilicity of the four ethoxy groups in the bis-phosphineamine PNP6, even though they share the same dithiocarbamate (DBODC) ligand (Figure 3).

These biodistribution studies on rats clearly evidenced that an appropriate molecular design enables the development of new cationic ^99m^Tc-radiopharmaceuticals with higher heart/liver ratios than those of [^99m^Tc]Tc-Sestamibi and [^99m^Tc]Tc-Tetrofosmin. The introduction of an alicyclic dithiocarbamate, instead of a linear one, significantly increases the myocardial uptake at an early injection time. Likewise, the incorporation of ether groups on the PNP ligand improve the heart uptake and the imaging properties of the complexes reducing the liver uptake and improving the hepatic clearance [43,44]. The crown ether was introduced in the chemical structure of the dithiocarbamate and bisphosphine ligand with the aim to modulate the lipophilicity of the radiotracer. In general, the lipophilicity of the radio tracer decreases as the number of ether linkages increases [45,46].

Other bidentate ligands, such as 2-mercaptopyridine oxide (MPO), were used to chelate the [^99m^Tc]TcN core in combination with bisphosphine [45,47]. The use of these bidentate chelating agents in place of the dithiocarbamates involves an easy modification of the lipophilic and biological properties of the cationic radiotracers of ^99m^Tc. For example, [^99m^Tc][TcN-MPO] and [^99m^Tc][TcN-DBODC5] have the same basic structure but differ in the chelating ligand donor of bidentate. Biodistribution studies in Sprague Dawley rats showed that [^99m^Tc][TcN-MPO] has a high initial heart uptake (2.45% ID/g at 5 min after injection), with long myocardial retention (2.44% ID/g at 120 min after injection) [48]. [^99m^Tc][TcN-MPO] myocardial uptake was higher than [^99m^Tc]Tc-Sestamibi, but lower than [^99m^Tc][TcN-DBODC5]. Liver clearance is rapid, resulting in an excellent heart/liver ratio. The heart/liver ratio of [^99m^Tc][TcN-MPO] at 30 min after injection is about 4-fold higher than that of [^99m^Tc]Tc-Sestamibi and twice that of [^99m^Tc][TcN-DBODC5]. After 120 min of injection, the heart/liver ratio of [^99m^Tc][TcN-MPO] increases to 27.60 and is thus 8-fold higher than that of [^99m^Tc]Tc-Sestamibi. Further evaluation in normal dogs showed that [^99m^Tc][TcN-MPO] had a blood clearance very similar to [^99m^Tc]Tc-Sestamibi, with <50% of starting activity at 1 min post-injection and <5% at 30 min post-injection. Whereas the images collected with ^99m^Tc-Sestamibi in dogs showed prolonged liver uptake, [^99m^Tc][TcN-MPO] liver uptake decreased very rapidly. SPECT images collected after the administration of [^99m^Tc][TcN-MPO] and [^99m^Tc]Tc-Sestamibi in canines with acute myocardial infarction indicated an early visualization (30 min after injection) of the perfusion defect with [^99m^Tc][TcN-MPO], but not with [^99m^Tc]Tc-Sestamibi. Based on the preclinical results, [^99m^Tc][TcN-MPO] was selected for clinical studies on humans. The whole-body images collected after 10 min from the administration of the radiotracer in healthy volunteers showed that, thanks to the fast radioactivity clearance, the myocardium was clearly separated from the left liver lobe. Its first-pass extraction fraction was higher than that of [^99m^Tc]Tc-Sestamibi but lower than that of [^99m^Tc]Tc-Tetrofosmin [5,49], and the rapid liver clearance allowed for both an early heart visualization in patients with CAD and a more precise determination of perfusion defects in the infer apical wall. For all these reasons, [^99m^Tc][TcN-MPO] is a good candidate to be used for patients with known or suspected CAD as an alternative to [^99m^Tc]Tc-Sestamibi. In fact, in these patients, the liver uptake makes it difficult to interpret the myocardium activity in the left and lower ventricular walls.

Studies conducted on the mechanisms of localization showed that the favorable pharmacokinetics of these [^99m^Tc][TcN-PNP] monocationic compounds result from the combined action of two major biochemical processes determined by their charge and lipophilia [44,50,51]. Myocardial absorption is the result of the accumulation of monocationic agents in the mitochondrial structures of myocytes, in response to a progressively rising driving potential from the outside of the cell to the inside of the mitochondrial matrix, while the lipophilic character of the compounds modulates their penetration into the cell membrane. The recognition of the complexes by the P-gp/MRP1 transporters then induces the rapid elimination of the activity from the liver to the intestine favoring the rapid lowering of the bottom activity, which in turn leads to high target/non-target ratios. This feature, from the point of view of the imaging properties, could help to reduce the duration of imaging protocols, allowing for a more accurate interpretation of the activity in the lower-left ventricular wall.

However, it should be considered that the favorable pharmacokinetic properties of the [^99m^Tc][TcN-PNP] monocationic compounds are somewhat negatively balanced by a relatively low first-pass myocardial extraction (about 60%)—a feature that associates them with the commercial agents, such as [^99m^Tc]Tc-Sestamibi and [^99m^Tc]Tc-Tetrofosmin—and by a varying amount of redistribution. The neutral compounds, [^99m^Tc]Tc-Teboroxime and [^99m^Tc][TcN(NOEt)_2_], show a higher (90%) extraction and can monitor blood flow better and over a wider range than tracers with a lower first-pass extraction. In addition, they are more suitable for a quantitative assessment of coronary stenosis under stress conditions [52].

The clinical trial results carried out on [^99m^Tc][TcN-DBODC5] show that, despite the good pharmacokinetics of this agent, which allows for better imaging quality and a higher clinical value for CAD detection than [^99m^Tc]Tc-Sestamibi, its clinical properties appear to be close to those observed for [^99m^Tc]Tc-Tetrofosmin. In terms of sensitivity, specificity, and diagnostic accuracy, the three radiopharmaceuticals seem to possess similar clinical properties [53].

## 5. Technetium-99m Radiopharmaceutical for MPI Based on the Tris-Carbonyl Core

**Figure 4:**
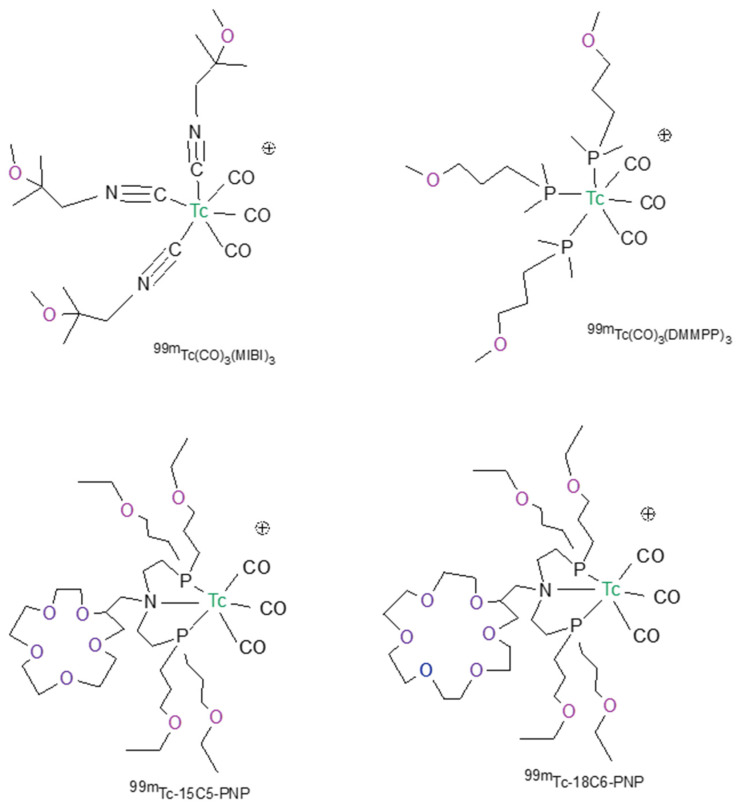
Chemical structures of the most representative [^99m^Tc]Tc(I)-tricarbonyl radiotracers for myocardial perfusion imaging.

The tris-carbonyl [99mTc][Tc(CO)3]+ core, largely used to develop target-specific radiopharmaceuticals [54,55], represents a different opportunity to prepare new [^99m^Tc]Tc(I)-tricarbonyl radiotracers for myocardial perfusion imaging. The [99mTc][Tc(CO)3]+ core has rich coordination chemistry that can be conveniently used to form radiotracers starting from the precursor [^99m^Tc][Tc(H_2_O)_3_(CO)_3_]^+^. In fact, in this metal fragment, the positions occupied by the three water molecules are labile and they can be easily substituted from appropriate ligands to form stable complexes [56]. Monodentate, bidentate, and tridentate ligands can be opportunely chosen to stably bind the technetium carbonyl core. The [^99m^Tc][Tc(L)_3_(CO)_3_] (L= dimethyl-3-methoxypropylphosphine (DMMPP) and 2-methoxy- isobutylisonitriles (MIBI)) (Figure 4) tracers were prepared by reacting the precursor [^99m^Tc][Tc(H_2_O)_3_(CO)_3_]^+^ with the monodentate DMMPP and MIBI ligands. The preparation of the precursor [^99m^Tc][Tc(H_2_O)_3_(CO)_3_]^+^ was carried out through a commercially available kit formulation. In Sprague Dawley rats, they showed a high myocardial uptake, with worse heart/liver and heart/lung ratios than of [^99m^Tc]Tc-Sestamibi, because of the slow hepatobiliary excretion [57]. A lower heart uptake was observed using the bidentate ligand (L-L) instead of the monodentate ligand L. In this case, it was assumed that the cationic [^99m^Tc][Tc(L-L)(CO)_3_]^+^ could form the neutral [^99m^Tc][Tc(L-L)(CO)_3_Cl] compound in vivo, due to the reaction with chloride ions present in the blood. These complexes have a low heart uptake and low hepatobiliary excretion [57]. On the contrary, the complexes [^99m^Tc][Tc(PNP)(CO)_3_]^+^ are highly stable monocationic compounds (Figure 4). In these complexes, the tridentate bis-phosphine confers stability and plays a fundamental role in maintaining the cationic nature of the radiotracer with a significant impact on the biodistribution of the tris-carbonyl technetium (I) compounds. A different number of oxygen atoms in the crown ether linked to the nitrogen atom of the diphosphine was introduced to balance the lipophilicity and modify the biodistribution characteristics of the cationic complex [58]. Among the radiotracers evaluated in the Sprague Dawley rats, ([^99m^Tc][Tc-15C5-PNP]) exhibited a high initial cardiac uptake and long myocardial retention. With regard to the cardiac uptake and heart-to-liver ratio, [^99m^Tc][Tc-15C5-PNP] is nearly identical to [^99m^Tc][TcN-DBODC5] [58]. It also showed a rapid elimination from the liver and lungs with a heart-to-liver ratio ~2.5 times better than that of [^99m^Tc]Tc-Sestamibi at 30 min post-injection. Planar imaging studies also showed that [^99m^Tc][Tc-15C5-PNP]P had a much better hepatic clearance profile than [^99m^Tc]Tc-Sestamibi.

**Figure 5:**
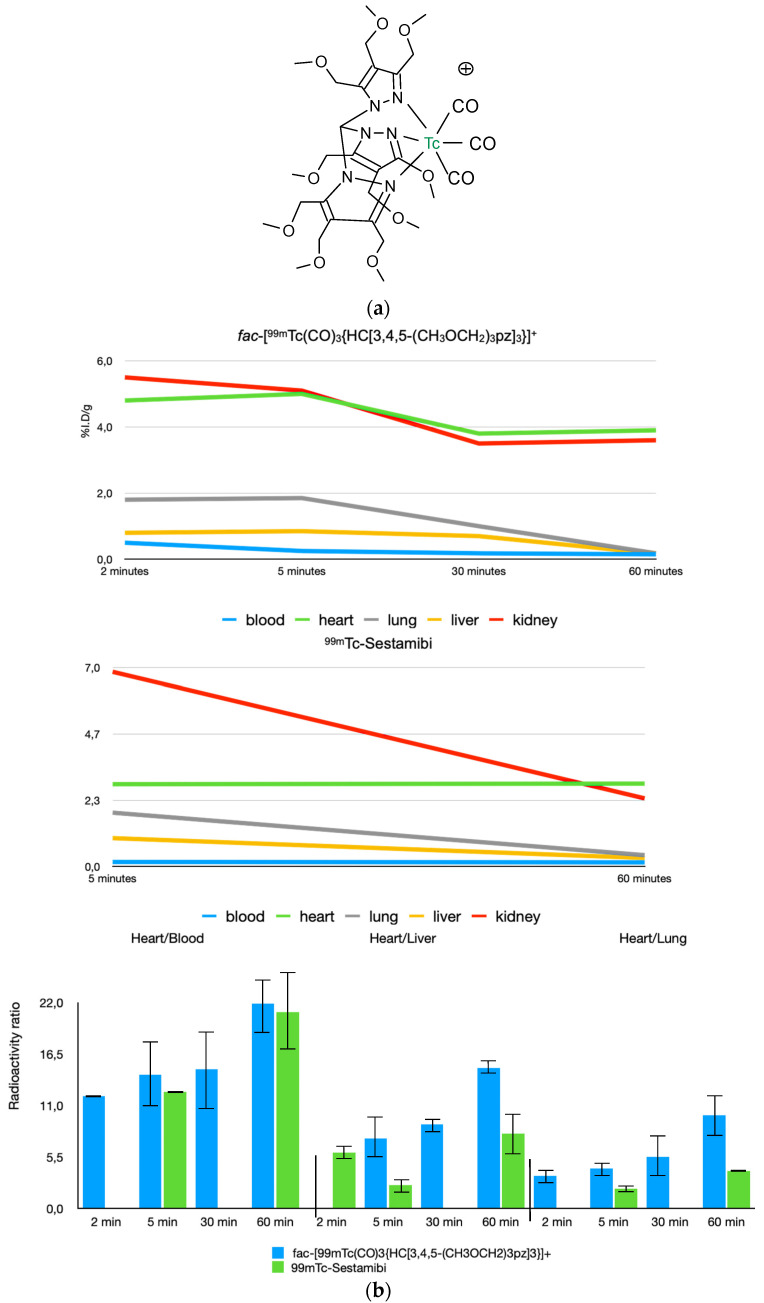
Chemical structure *fac*-[^99m^Tc][Tc(CO)_3_{HC[3,4,5-(CH_3_OCH_2_)_3_pz]_3_}]^+^ (**a**) and biodistribution data in rats compared to [^99m^Tc]Tc-Sestamibi (**b**) [59].

A different tridentate ligand containing the functionalized tris(pyrazolyl) methane chelators was used to develop promising heart imaging agents with the [99mTc][Tc(CO)3]+ core [59]. The functionalization of the tris(pyrazolyl)methane chelators with two or three methoxymethyl groups at the azole rings allowed for the modulation of the biodistribution profile and pharmacokinetics of the respective ^99m^Tc(I) tricarbonyl complexes. In particular, the *fac*-[^99m^Tc][Tc(CO)_3_{HC[3,4,5-(CH_3_OCH_2_)_3_pz]_3_}]^+^ complex with 3 methoxymethyl groups at the azole rings was chosen for imaging studies in rats due to its enhanced heart/liver and heart/lung ratios, in comparison with [^99m^Tc]Tc-Sestamibi, namely at earliest post-injection times. It exhibits a significantly high and stable heart uptake (Figure 5) associated with a very fast liver clearance, and represents, to date, to the best of our knowledge the most promising [^99m^Tc]Tc(I)-tricarbonyl complex as a myocardial imaging agent.

## 6. Development and Application of High-Resolution CZT Cameras

Detectors traditionally used in SPECT are based on scintillation, a phenomenon that occurs as a result of the interaction of ionizing radiation with a scintillator material (in general, a NaI crystal), resulting in the emission of light by fluorescence or phosphorescence. They allow for the simultaneous exploration of large body areas and are therefore used for the acquisition of static, dynamic, and tomographic scintigraphic images. For decades, the SPECT survey tools have remained essentially unchanged and, due to the lower potentials, the lack of technological innovation seemed to be destined to be totally replaced by the advent of PET tomography.

Thanks to the recent technological progress, the introduction of the cadmium-zinc-telluride (CZT) semiconductor detectors, as an alternative to traditional NaI crystal-based systems, has completely changed the scenario. The high atomic number of these materials allows for a good detection efficiency even with very thin thicknesses. Thanks to the high absorbing capacity, the energy range of radiation to be detected is wide, from tens of KeV to some MeV. As a result of the direct conversion of photons into electrons, there is the collection of a greater number of charges to the electrodes, which results in less uncertainty in the energy of photons. CZT semiconductors work at room temperature and can process 10 million photons/second/mm^2^, boasting the highest energy resolution among all the commercially available scintillators [60]. Additionally, the hardware implementation of this system consists of the utilization of semiconductor modules on a column of tungsten collimators rather than lead. Therefore, lead elimination from the detector suite avoids the potential X-ray generation, which can degrade the image quality. The use of these systems allowed us to reduce the acquisition times and the absorbed doses with the simultaneous increase in photon sensitivity and spatial resolution [61].

The development of cadmium-zinc-tellurium detectors for medical investigations is actually focused on the discovery of new applications, mainly in the cardiac and oncological fields. In particular, in this scenario, various devices have been designed and built, primarily dedicated to the study of the myocardium by applying different geometric configurations. The main products on the market are represented by the D-SPECT and VERITIONTM cameras (Spectrum Dynamics, Palo Alto, CA, USA) and by the Discovery NM 530c and NM/CT 670 CZT NM/CT 570c cameras (GE Healthcare, Haifa, Israel). All of this equipment is capable of performing dynamic SPECT in order to assess myocardial blood flow (MBF) [62].

In conclusion, the introduction of CZT technology for SPECT imaging, offering significant improvements in terms of speed, spatial sensitivity, and resolution in comparison to conventional tomographic gamma cameras, opened up new perspectives in cardiac imaging studies. This not only may improve the performance of actually available tracers, but could also lead to the rediscovery of some original tracers, previously abandoned, such as, for example, [^99m^Tc]Tc-Teboroxime and the development of new improved tracers, such as [^99m^Tc]Tc-3SPboroxime. The high initial absorption of the heart, combined with longer myocardial retention and high heart/background ratios, suggests that [^99m^Tc]Tc-3SPboroxime may become a candidate for future clinical translation through the new ultrafast CZT-SPECT detectors. Using these new SPECT cameras, a myocardial tomography can be realized in less than 5 min with half of the dose normally administered with conventional SPECT imaging devices, and, moreover, simultaneous dual-tracer protocols can be applied [63,64,65,66,67,68].

## Figures and Tables

**Table 1:** Properties of selected ^99m^Tc-labeled MPI agents.

	[^99m^Tc]Tc-Sestamibi	[^99m^Tc]Tc-Tetrofosmin	[^99m^Tc]Tc-Teboroxime	[^99m^Tc]Tc-3SPboroxime
Chemical characteristics	cationic	cationic	neutral	neutral
Kit formulation	available	available	available	available
Myocardial uptake at rest (%)	1	1.2	3–4 *	4–5
Heart/to liver ratio 15–20 min post-injection at rest	0.5	0.8	negligible	0.8
First pass extraction (%)	65	54	88	-
Redistribution	negligible	none	significant	-
Excretion	hepatobiliary	renal and hepatobiliary	hepatobiliary and renal	

* soon after injection.
